# A method for near full-length amplification and sequencing for six hepatitis C virus genotypes

**DOI:** 10.1186/s12864-016-2575-8

**Published:** 2016-03-17

**Authors:** Rowena A. Bull, Auda A. Eltahla, Chaturaka Rodrigo, Sylvie M. Koekkoek, Melanie Walker, Mehdi R. Pirozyan, Brigid Betz-Stablein, Armin Toepfer, Melissa Laird, Steve Oh, Cheryl Heiner, Lisa Maher, Janke Schinkel, Andrew R. Lloyd, Fabio Luciani

**Affiliations:** School of Medical Sciences, Faculty of Medicine, University of New South Wales, Sydney, 2052 Australia; Department of Medical Microbiology, Section of Clinical Virology, Academic Medical Center, Public Health Service, Amsterdam, The Netherlands; The Kirby Institute, University of New South Wales, Sydney, Australia; Pacific Biosciences, Menlo Park, CA USA

## Abstract

**Background:**

Hepatitis C virus (HCV) is a rapidly evolving RNA virus that has been classified into seven genotypes. All HCV genotypes cause chronic hepatitis, which ultimately leads to liver diseases such as cirrhosis. The genotypes are unevenly distributed across the globe, with genotypes 1 and 3 being the most prevalent. Until recently, molecular epidemiological studies of HCV evolution within the host and at the population level have been limited to the analyses of partial viral genome segments, as it has been technically challenging to amplify and sequence the full-length of the 9.6 kb HCV genome. Although recent improvements have been made in full genome sequencing methodologies, these protocols are still either limited to a specific genotype or cost-inefficient.

**Results:**

In this study we describe a genotype-specific protocol for the amplification and sequencing of the near-full length genome of all six major HCV genotypes. We applied this protocol to 122 HCV positive clinical samples, and had a successful genome amplification rate of 90 %, when the viral load was greater than 15,000 IU/ml. The assay was shown to have a detection limit of 1–3 cDNA copies per reaction. The method was tested with both Illumina and PacBio single molecule, real-time (SMRT) sequencing technologies. Illumina sequencing resulted in deep coverage and allowed detection of rare variants as well as HCV co-infection with multiple genotypes. The application of the method with PacBio RS resulted in sequence reads greater than 9 kb that covered the near full-length HCV amplicon in a single read and enabled analysis of the near full-length quasispecies.

**Conclusions:**

The protocol described herein can be utilised for rapid amplification and sequencing of the near-full length HCV genome in a cost efficient manner suitable for a wide range of applications.

**Electronic supplementary material:**

The online version of this article (doi:10.1186/s12864-016-2575-8) contains supplementary material, which is available to authorized users.

## Background

Hepatitis C virus (HCV) is a significant human pathogen affecting nearly 3 % of the world’s population, and is a leading cause of chronic liver diseases including cirrhosis and hepatocellular carcinoma [1]. HCV is a member of the *Flaviviridae* family and has a single stranded RNA genome that is 9.6 kb in length with positive polarity. The genome contains a single open-reading frame and encodes a precursor polyprotein of approximately 3010 amino acid residues. Within an infected cell, this polyprotein is processed by cellular and host proteases to yield ten structural (core, E1 and E2) and non-structural (p7, NS2, NS3, NS4A, NS4B, NS5A and NS5B) proteins [2]. The viral RNA-dependent RNA polymerase, or NS5B, is a key enzyme in the HCV replication complex within an infected cell, and is responsible for the production of nascent genomes for packaging into new virions. The polymerase is highly error-prone [3], a feature common to many RNA viruses, and as a result HCV exists as seven distinct genotypes (GT1-7), which differ by up to 35 % at the nucleotide level [4]. Within each genotype, viruses have been further classified into subtypes (1a, 1b, 1c, etc.) with about 20 % inter-subtype nucleotide divergence [5]. The genotypes are unevenly distributed across the globe, with genotypes 1 and 3 being the most prevalent [6], and genotype 7 being the rarest having only been detected once in Canada from a Central African immigrant [7].

With the advent of direct-acting antivirals (DAA) for the treatment of HCV infections, there is a need to monitor the emergence of resistance-associated variants before and after treatment. While testing specific regions of the HCV genome using both consensus and next generation sequencing (NGS) has enabled such monitoring in DAA therapies, including agents targeting NS3, NS5A and NS5B [8, 9], the emerging DAA combination regimens emphasise the necessity to simultaneously screen multiple genes of the viral genome in a simple, cost-effective manner. Furthermore, there is a need to detect whether individual viral variants carry multiple polymorphisms conferring resistance against all DAAs in a combination regimen, therefore increasing the chances of viral persistence against such therapies. In the past, such analysis was possible only by cloning and consensus sequencing. However, as the NGS technologies continue to improve read length (20 kb with PacBio RS and 300 bp paired-end for Illumina) the range for covariant studies continues to increase.

In addition to monitoring viral variants associated with drug resistance, methods for the molecular amplification and sequencing of HCV RNA have also been instrumental in characterising HCV infections, including studies seeking to understand virus transmission and within-host evolution [10–12]. Previously, these methods focused on specific regions of the HCV genome, or analysed the entire genome in separate fragments, which is laborious, cost-prohibitive and leads to analysis issues, including uneven coverage due to amplicon pooling, artificial recombinants during genome assemby and multiple PCR primer bias. Therefore, there is a need for sensitive methods that can amplify the entire viral genome from all GTs in clinical samples. A few reports have described the amplification of near full-length HCV genomes, however these were either limited by the sensitivity of the methodology or coverage of all major genotypes [13–15]. Recently, RNA sequencing technology has been applied to sequence HCV in a non-specific manner [15]. While these methods offer the advantage of reduced primer bias and reduced upstream labour, they require increased labour in data analysis, have reduced sensitivity and increased overall cost per sample [15].

Here, we describe a simple method which allows sensitive amplification of near full-length HCV genomes from GT 1 to 6. Using this method, 90 % (*n* = 121) of a set of HCV-infected clinical samples were successfully amplified and sequenced using an NGS approach. As a proof of principle, the method was also applied to amplify and sequence near full-length HCV genomes from two subjects co-infected with multiple genotypes. The ability to generate near full-length quasispecies sequence was tested with one amplicon using the PacBio RS II platform.

## Methods

### Cohort

Stored plasma samples positive for HCV GT1, 2, 3 and 6 were made available from the Hepatitis C Incidence and Transmission Study in prisons and community cohort (HITS), which is a prospective cohort of HCV seronegative and HCV RNA negative subjects in New South Wales, Australia [16]. Stored plasma samples positive for HCV GT 4 and 5 were from Academisch Medisch Centrum (AMC) patients identified with HCV infection. In all subjects, HCV infection was confirmed by detection of HCV-specific antibody and RNA. In the HITS cohort, HCV antibody testing was performed using the qualitative Abbott ARCHITECT anti-HCV chemiluminescent microparticle immunoassay (Abbott Diagnostics, Abbott Park, IL, USA). For the AMC patients HCV antibody testing was performed using the AxSYM HCV 3.0 serology test (Abbott Laboratories, Abbott Park, IL, USA). For all subjects quantitative HCV RNA detection was performed with the COBAS AmpliPrep/COBAS TaqMan HCV assay (Roche, Branchburg, NJ, USA; lower limit of detection 15 IU/ml).

### Ethics statement

For the HITS samples ethical approvals were obtained from Human Research Ethics Committees of Justice Health (reference number GEN 31/05), New South Wales Department of Corrective Services (reference number 05/0884), and the University of New South Wales (reference numbers 05094, 08081), all located in Sydney, Australia. Written informed consent was obtained from the participants. For the Dutch samples, the study was performed according to the Dutch FEDERA code of conduct for responsible use of human tissue and medical research 2011.

### Genotyping and detection of multiple infection

Genotype determination and detection of multiple HCV genotype infection was performed on a region of the core as previously described [17].

### Primer design

Full-length genome sequences representing each of the six HCV genotypes were downloaded from GenBank and used for primer design. This included 116, 28, 50, 66, 11 and 7 different strains for GTs 1–6 respectively. To choose primer binding sites we manually scanned the 3′ end of the genome for regions >20 bp with a minimum of 90 % identity. To improve sequence identity of the primer with the viral variants, degenerates bases were inserted where needed. To improve binding efficiency degenerate bases were not added to the last 3 nucleotides at the 3′ end of the primer and where possible primers had C’s or G’s at the 3′ base.

### JFH-1 RNA T7 transcripts

T7 RNA transcripts for full-length cell culture derived HCV genotype 2a variant, JFH-1, were generated as previously described [18].

### RNA concentration by ultracentrifugation

For concentration of HCV from plasma samples, 0.5–1 ml of plasma was thawed and centrifuged at 1,500 g for 10 min at 4 °C. Supernatants were transferred and centrifuged at 120,000 g for 1.5 h at 4 °C after which the pellet was re-suspended in 140 μl of 1× PBS. Viral RNA was then extracted from the sample as outlined below.

### RNA extraction

Viral RNA was extracted from 140 μl of plasma using the QIAmp Viral RNA kit according to manufacturers’ instructions (Qiagen, Chadstone Centre, Vic, Australia), with the following modifications: Ambion® linear acrylamide (5 μg/extraction, Life Technologies) was used instead of the carrier RNA provided in the kit; sample lysis was performed by inverting tubes instead of vortexing; and the speed of centrifugation was reduced to 3,421 g for all steps except the final wash which was carried out at 6,082 g. Finally, RNA was eluted in 50 μl of RNA Storage Solution (1 mM sodium citrate pH 6.4, Life Technologies) and stored at −80 °C.

### Reverse transcription

Near full-length HCV cDNA was synthesized from viral RNA using the SuperScript III (SIII) First-Strand Synthesis System (Life Technologies) and a pan-genotype primer (oligo dA_20_, Table 1). Before commencing reverse-transcription (RT), 7 μl of RNA, 1 μl of 10 μM primer and 1 μl of 10 mM dNTPs (Promega, Alexandria, NSW, Australia) were mixed and incubated in a thermocycler (T100™ BioRad, Gladesville, NSW, Australia) at 65 °C for 5 mins and then placed immediately on ice for 1 min. RT was then initiated with the addition of RT reaction mix to a 20 μl final volume at a final concentration 1x RT buffer, 5 mM MgCl_2_, 1 M Betaine (Sigma, Sydney, NSW, Australia), 1 μl of RNAseOUT (Life Technologies) and 9 U of SIII RT. Cycling conditions were 49 °C for 65 min, followed by 85 °C for 5 min. Two units of RNaseH (Life Technologies) were then added to each reaction before a final incubation at 37 °C for 20 mins. cDNA samples were held at 12 °C before proceeding to the PCR reaction.

**Table 1:** Primers for near full-length HCV amplification

Region	Round	Sense	GT	Primer name	Sequence (5′-3′)^a^	Genome binding position^b^	Published
3′UTR	RT	-	All	vir7	AAAAAAAAAAAAAAAAAAAA	9418-9486	[13]
5′UTR-NS5B	Outer	+	All	KY80	GCAGAAAGCGTCTAGCCATGGCGT	68-91	[28]
		-	1	Vir45a	CCAGCGGGGYCGGGCVYGAGACA	9262-9314	Unpublished
		-	2	hep323	GGAGTGTASCTARTGTGTGCCGCT	9378-9401	[11]
		-	3	hep234b	TGGAGTGTTATCYTACCAGC	9378-9397	[11]
		-	4	GEN4.R1	TCGGGCAYGRGACAYGCTGTGATAAATG	9278-9305	[11]
		-	5	GEN5.R1	TCGGGCACGGGACATGCTGTGATAAATG	9278-9305	Unpublished
		-	6	Vir65	CGRGCCYGGGACACGCTGTG	9285-9304	Unpublished
5′UTR-NS5B	Inner	+	All	hep21b	GAGTGTYGTRCAGCCTCCAGG	98-118	[20]
		-	1,2,3,6	hep296	CGGGCAYGAGACASGCTGTGATAWATGTC	9276-9304	[11]
		-	4	GEN4.R2	TCTCCCCCGCCRGCGCCYACCGTRAACC	9250-9277	Unpublished
		-	5	GEN5.R2	TCCCCCCCGCCRGCGCCAACGGTRAACC	9250-9277	Unpublished

^a^For degenerate primers, B = C or G or T, H = A or C or T, M = A or C, N = A or C or G or T, R = A or G, S = C or G, W = A or T, V = A or C or G, D = A or G or T, Y = C or T

^b^Genome binding position with reference to HCV GT1a strain, H77, GenBank accession AF009606

For optimisation experiments, the yield of near full-length amplicons was compared between two different SuperScript enzymes II and III (as above) (Invitrogen). For the SII enzyme the RT reaction was performed as described above, except the SuperScript II buffer and SuperScript II enzyme was substituted into the assay. JFH-1 RNA was used as template.

### Comparison of different polymerase enzymes for efficient near full-length HCV amplification

Using full-length JFH-1 cDNA as template, transcribed as described above, two different polymerase combinations were tested for their efficiency in amplifying near full-length genome and a 4 kb fragment from the 5′ end of the HCV genome in a single round. Primers KY80 and vir45a (Table 1) were used for near full-length amplification, while vir45a was replaced by hep344 [11] for the 4 kb fragment. The two enzymes tested in parallel were KlenTaq LA (Clontech) and KlenTaq DNA polymerase (AB Peptides, St. Louis, MO) that was used at a ratio of 2 to 1 with *pfu* DNA polymerase (Stratagene, La Jolle, CA). The reaction conditions of the KlenTaq LA are detailed in the next paragraph. The reaction conditions for the KlenTaq/*pfu* mix were performed as described by Zhang et al. [13] with the exception that the PCR cycling conditions were the same as those performed with the KlenTaq LA reaction, as described below.

### PCR of near full-length HCV genome

First round PCR was performed using 5 μl of cDNA in a 50 μl PCR reaction containing 1× KlenTaq LA buffer (Clontech), 1.2 M betaine, 200 μM of each dNTP, 0.2 μM of the forward primer KY80, 0.2 μM of the genotype specific reverse primer (Table 1) and 1U of KlenTaq LA enzyme. PCR was performed at 94 °C for 2 min, 10 cycles at 94 °C for 30 s, 55 °C for 20 s, and 68 °C for 11 min, followed by an additional 20–25 cycles at 94 °C for 30 s, 57 °C for 20 s, and 68 °C for 10:30 min (+20 s/cycle), with a final extension for 5 min at 68 °C.

The nested round of PCR was performed with 5 μl of first round product in a 50 μl reaction as described for the first round, except 0.2 μM of hep21b was used as the forward primer and 0.2 μM of a nested genotype-specific reverse primer (Table 1). PCR was performed at 94 °C for 2 min, 10 cycles at 94 °C for 30 s, 58 °C for 20 s, and 68 °C for 10:30 min, followed by an additional 20–25 cycles at 94 °C for 30 s, 60 °C for 20 s, and 68 °C for 10:30 min (+20 s/cycle), and then followed by a final extension for 5 min at 68 °C.

Following positive identification of a band of the correct size, which was approximately 9206 bp (according to HCV GT1a strain H77, GenBank accession AF009606) (Additional file 1: Figure S1), the products were purified using the Agencourt AMPure XP beads (Beckman Coulter, Lane Cove, NSW, Australia) according to the manufacturers instructions, eluted in nuclease-free water and stored at -80C.

### Real-time nested PCR

HCV RNA was quantified by real-time PCR using a Bio-Rad MyiQ Single-Color real-time PCR detection system (Bio-Rad, CA) as described previously [19] with the exception that primers hep21b (Table 1) and hep22 were used [20] and near full-length cDNA generated as described above, was used as template. PCR band intensities were also quantified by densitometry using ImageJ (version 1.46r).

### Illumina sequencing

**Fig. 1:**
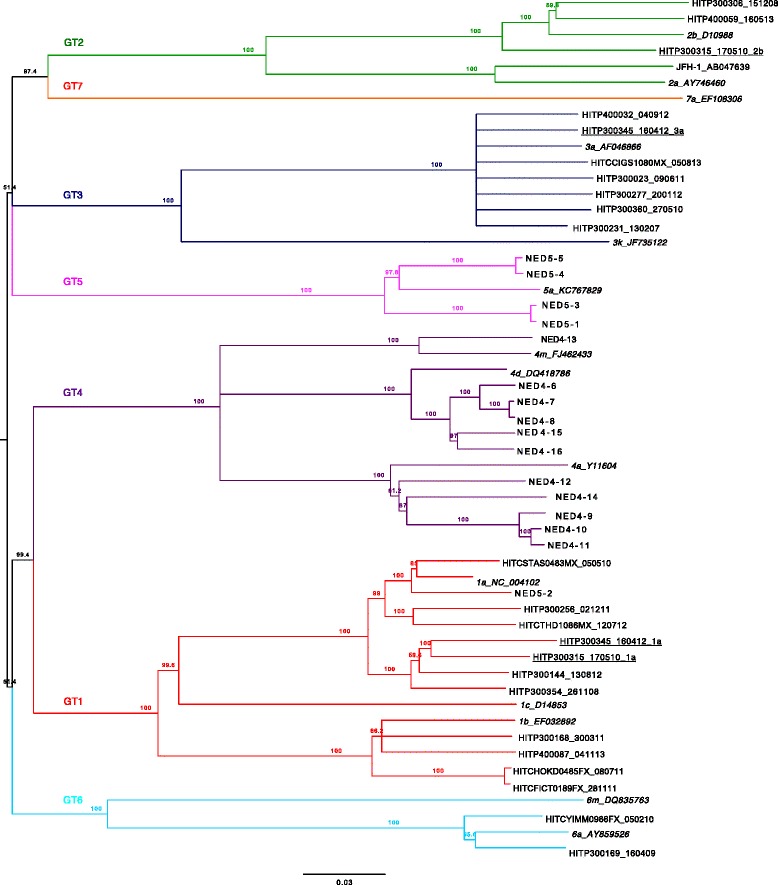
Phylogenetic analysis of the near full-length HCV genomes from 37 clinical samples. The phylogenetic tree was generated using the Neighbour-Joining method implemented in Geneious package version 8 [22]. Reference genomes are italicised with their subtype listed first, followed by their GenBank accession number. Sequences that belonged to clinical samples with known mixed infections are underlined (GT1a/3a in HITP300345, and GT1a/2b in HITP300315). Branches are colored according to HCV genotype. The branch lengths are proportional to the evolutionary distance between sequences and the distance scale, in nucleotide substitutions per position, is shown

Libraries were prepared from the amplicon using either the Nextera XT or TruSeq Nano DNA Library Prep Kits (Illumina) before sequencing using a MiSeq Benchtop Sequencer generating 2 × 300 bp length paired-end reads (v3 kit). NGS reads were aligned using Bowtie 2 [21] implemented in Geneious package version 8 [22]. Reads were aligned against a panel of full-length reference genomes that were obtained from GenBank to represent GTs 1–7 (Fig. 1), and consensus sequences were generated from the aligned contigs. Mixed infection was identified by the generation of multiple contigs against two or more reference genomes. In these situations, a consensus genome sequence was generated for each contig.

### PacBio sequencing

One of the full-length amplicons, RIMM, was selected for generating >9 kb sequence reads with PacBio SMRT sequencing. Unique PacBio barcodes were ligated to the amplicon and approximately 0.8 fmoles of the product was then sequenced via 240-min movies on one SMRT Cell using P6-C4 chemistry on a PacBio RS II sequencer (Menlo Park, Pacific Biosciences, California, USA). Genome filtering, assembly and reassembly were performed using tools within SMRT Analysis v2.3. SMRTbell adapter sequences were removed and circular consensus sequence (CCS) reads with a minimum of 2 full passes of the full amplicon (ie. >18 kb) were selected for further analysis. These reads were *de novo* assembled using VICUNA [23].

### Haplotype reconstruction of near full-length HCV quasispecies using PacBio SMRT sequencing

PacBio RS II reads were analysed using haplotype reconstruction analysis, as previously performed [11]. Briefly, haplotype analysis was performed to correct for random technical errors using the software ShoRAH [24]. Only unique haplotypes and their estimated frequency of occurrence within the quasispecies population were reported. Indels identified in homopolymeric regions were manually replaced with the consensus sequence.

### Phylogenetic analysis

The 39 Illumina generated consensus genomes from the 37 clinical samples and the cell culture derived test sample JFH-1 were aligned against representative subtypes from the GT1 to 7 reference genomes with the alignment tool MUSCLE, implemented in Geneious package version 8 [22]. Bootstrapped trees (500 data sets) were constructed using the Neighbour-joining method, also implemented in Geneious package version 8 [22]. Phylogenetic analysis with the PacBio generated haplotypes was performed as described above.

## Results and discussion

### Primer design

Full-length genome alignments representing all six HCV GTs were used to assess previously published and novel primer sites for pan-genotypic potential. The previously published oligo dA_20_ primer was selected for the RT step due to its pan-genotypic quality [13]. It was possible to identify pan-genotypic primers for both the forward outer (KY80) and nested (hep21b) primers, but genotype-specific primers were designed for the reverse primer pairs, outer and nested (Table 1).

### RNA extraction optimisation

For the RNA extraction, the QIAmp viral RNA mini kit was used but several modifications were made to the manufacturer’s protocol. These modifications are outlined in detail in the methods section. In brief, the carrier RNA was substituted with 5 μg of linear acrylamide, as previously recommended for other RNA viruses, HIV, RSV and WNV [25]. Elution of purified RNA in RNA Storage Solution was essential for optimal results, particularly after long-term storage and repeated freeze-thaw cycles. Successful amplification of near full-length genomes from stored RNA samples was reproducible six months after initial extraction. The centrifugation speeds were also reduced as this was shown to reduce shearing of RNA. Also as successful amplification of the full genome requires intact genomes, mixing of sample by pipetting and vortexing was limited.

### cDNA optimisation

As part of the optimisation process for cDNA generation, RT enzymes, reaction conditions and thermocycling conditions were examined using JFH-1 RNA as template and near full-length amplicon yield as a measure of success. For the RT enzyme analysis, two RT enzymes, SuperScript II and III were compared, with the SIII enzyme generating a 2.6-fold higher yield. For the RT reaction conditions, it has been previously shown that the addition of Betaine at 1 M concentration is optimal in similar assay conditions [26]. Therefore, we tested the effect of Betaine at a final concentration of 1 M on amplicon yield. The cDNA reactions containing 1 M Betaine generated an amplicon with a 9.5-fold increase in yield compared to cDNA conditions with no Betaine (data not shown). In regard to thermocycling conditions, previous reports had indicated optimal cDNA generation of full-length HCV with an extension time of 2.5 h with varying extension temperatures [13]. In our study, optimal results were obtained with a constant extension temperature of 49 °C for 65 mins. It was also noted that more consistent results were obtained when the PCR proceeded immediately after the RT step had finished. Storage of cDNA at either −20 °C or −80 °C greatly reduced the success rate in generating near full-length amplicons.

### PCR optimisation

For PCR optimisation, two different KlenTaq mixes were compared. The polymerase combination of KlenTaq and *Pfu* at a ratio of 2 to 1, as outlined by Zhang et al. [13], was compared using either the thermocyling conditions published by Zhang et al. or as recommended for the other commercial KlenTaq mix, KlenTaq LA (see Methods). Both of these conditions were used for the KlenTaq/*Pfu* mix and compared directly to the commercial polymerase mix of KlenTaq LA (Clontech) that only used the conditions recommended by Clontech. The three different enzyme/assay conditions were tested for amplification efficiency using primer sets that would either generate a 9 kb or 4 kb fragment. No amplicons for either the 4 or 9 kb amplicon were generated following the reaction and cycling conditions nominated by Zhang et al. [13]. For the remaining two enzyme/assay conditions, the yield of the ~9 kb fragment was increased by 5.5-fold with the commercial mix, KlenTaq LA compared to the KlenTaq/*Pfu* mix run with the same cycling conditions (data not shown). A 4 kb region covering the 5′ end of the genome was also amplified with both polymerase mixes using Clontech’s conditions and only a slight increase, 1.3-fold, in amplicon yield of the 4 kb fragment was observed for the KlenTaq LA polymerase mix. Overall, these results demonstrated the superior performance of the KlenTaq LA mix for the near full-length amplicon, and the kit was adopted for further optimisation.

Generally, the protocol provided with the KlenTaq LA enzyme from Clontech was found to be optimal with the only modification being the addition of Betaine. After the addition of 5 μl of cDNA to the PCR assay the final Betaine concentration was 1.3 M, which is the recommended concentration for the KlenTaq enzyme.

The efficiency of amplicon generation, in terms of yield of amplicon and reduced non-specific amplification was improved if the RT and PCR primers were aliquoted into small batches with no subsequent freeze/thaw cycles before use. Freeze/thaw rounds of dNTPs did not appear to affect the generation of amplicons.

### Detection limit

To determine the detection limit of the nested PCR assay, a representative cDNA sample for GT1, 2 and 3 was generated. The copy number of near full-length cDNA transcripts was determined by real-time PCR targeting the 5′UTR of the genome. The cDNA samples were then serially diluted and the limit of detection assessed. The assay had a detection limit of 1–3 copies of HCV near full-length cDNA per reaction. For this assay, we calculated the detection limit using near full-length cDNA transcripts. This was chosen as there are many independent variables that impact the generation of full-length cDNA transcripts from a HCV positive plasma sample [27]. For instance, plasma collection protocols, date of collection and storage conditions - particularly in regards to time delays post collection and freeze/thaw occurrence can result in RNA degradation, and subsequently impact the generation of full-length cDNA transcripts. Traditionally, methods used to determine viral RNA genome copies target a small region of the genome and are resilient to RNA degradation and therefore not a good indicator of the integrity of full length viral RNA genomes.

### Robustness of near full-length amplification

**Fig. 2:**
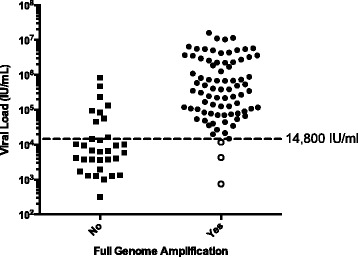
Sensitivity of the near full-length assay tested on HCV positive clinical samples. The success rate of amplifying near-full length genomes in relation to viral load is shown for 122 samples. Eighty-six of the 95 HCV positive samples with a viral load greater than 14,800 IU/ml were successfully amplified (90.5 % success rate). All 27 samples with a viral load below 14,800 IU/ml failed to amplify, however, 10 of these samples were retested following concentration by ultracentrifugation, of which three (30 %) were successfully amplified (hollow circles)

To test the robustness of the assay on clinical samples, 122 HCV samples with known genotypes were tested for near full-length nested RT-PCR amplification. The viral loads ranged from 317 to 1.59 × 10^7^ IU/ml. The lowest viral load that was able to generate a near full-length amplicon was 14,853 IU/ml. All 27 samples with a viral load below 14,800 IU/ml failed to amplify (Fig. 2). Another 9 samples with viral loads above 15,000 IU/ml also failed to amplify. Therefore, sensitivity testing indicated that the PCR was robust with 90.5 % of the samples being successfully amplified with a viral load >14,800 IU/ml (equivalent to 39,960 copies/ml) (Fig. 2). Analysis of the data stratified by genotype, revealed that GT2b had the lowest success rate of 75.0 %, followed by GT3 and GT1 at 87.1 % and 90.9 %, respectively (Table 2). All remaining genotype samples amplified with a 100 % success rate, although it should be noted that due to these other genotypes being less common in our cohorts their representation in the sample set was lower (Table 2). A representative set of amplicons (*n* = 37) for each genotype were sequenced on the Illumina MiSeq platform. Phylogenetic analysis was used to confirm the success of the assay in amplifying GTs 1–6 (Fig. 1).

**Table 2:** Near-full genome amplification success rate by genotype

HCV GT	Number of samples tested (%)^a^	
	Successful	Failed
1a	30 (90.9)	3 (9.1)
1b	6 (100)	0 (0)
2a^b^	1 (100)	0 (0)
2b	6 (75)	2 (25)
3a	27 (87.1)	4 (22.9)
4a,d,m	11 (100)	0 (0)
5a	4 (100)	0 (0)
6a	3 (100)	0 (0)

^a^Only includes samples with viral loads greater than 14,800 IU/ml

^b^This was cell cultured derived JFH-1 and was not included in the success rate

### Ultracentrifugation

For samples with low viral loads (less than 15,000 IU/ml) sensitivity of detection could be improved by concentrating the virus from the plasma by ultracentrifugation. In this study we selected 10 samples that had detectable RNA with the real-time PCR assay that targeted a 175 bp region in the 5′UTR, but could not initially be amplified with the near full-length assay. For these samples, RNA was extracted from 1 ml of plasma after ultracentrifugation. Upon retesting, a near full-length amplicon was generated for 3 of the 10 samples that had previously failed. While ultracentrifugation of larger sample volumes improved the sensitivity of near full-length amplification, we observed that simply increasing the plasma volume from which virus was extracted without ultracentrifugation did not. We speculate that either the additional spin duration, total extraction time or additional PCR inhibitors from the increased plasma volume may account for the reduced RNA extraction efficiency when the plasma volume extracted through the QIAmp column is increased.

### Near full-length sequencing on PacBio

**Fig. 3:**
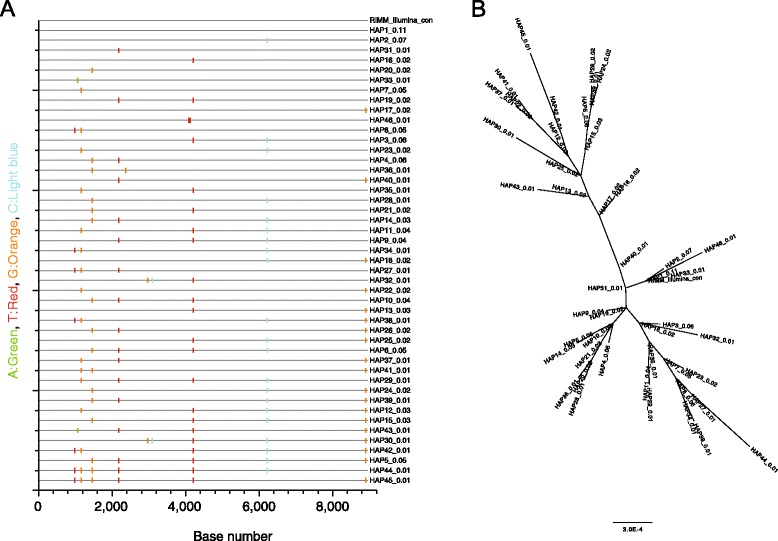
Analysis of the HCV quasispecies utilising near full-length reads with the PacBio RS platform. Individual sequences reads greater than 9 kb were generated and aligned for an HCV infected individual, RIMM. Panel (**a**). Highlighter plot (www.hiv.lanl.gov/content/sequence/HIGHLIGHT/highlighter.html) representing 45 unique haplotypes that were detected from sequence reads. The name of each haplotype shows the frequency of occurrence within the population (e.g. HAP_0.11). The master sequence on the top of the plot is the consensus genome sequence obtained from the Illumina sequences. Panel (**b**). Phylogenetic tree showing the genetic relationship between near-full length quasispecies obtained from PacBio reads

To test whether near full-length sequence reads could be generated from the HCV amplicon, the sample RIMM was selected for sequencing on the PacBio RS II. A total of 2,664 reads had a minimum of two full passes of the 9.2 kb amplicon (>18 kb in length) and were used for further analysis. The near-full length PacBio reads were then error corrected via haplotype reconstruction analysis [11]. This analysis showed 45 distinct variants with the most common having a frequency of occurrence of 11 % (HAP1_0.11, Fig. 3), which was identical to the consensus genome sequence generated from the Illumina data.

The sensitivity of PacBio to detect low frequency mutations was compared to the data generated by Illumina from the same amplicon. Both sequencing platforms detected all 11 SNPs with a frequency greater than 7 % (data not shown). The PacBio reads after cleaning with ShoRAH only detected 4.2 % (3 of a total of 71) of the SNPs detected with the Illumina platform with a frequency below 7 %. However, it should be noted that the genome sequence coverage generated by Illumina was about five-fold higher, with a mean coverage of 11,363 reads/site, respectively and likely accounts for the discrepancy in calling low frequency events.

Phylogenetic analysis of the near-full length haplotypes was performed to understand the relationship of the quasispecies in the acutely infected HCV subject. The analysis depicted the presence of two distinct sub-populations that carry most of their diversity in the structural region of the genome (position 114–2505, Fig. 3a). This analysis confirms the applicability of this approach to perform near-full genome quasispecies analysis.

### Multiple infection

**Fig. 4:**
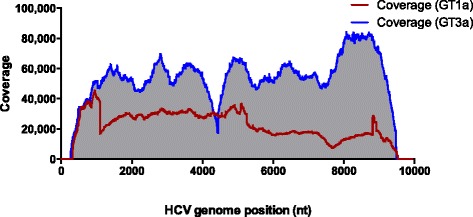
Subject HITSP300345_160412 was identified as being infected with two HCV genotypes (GT), GT1a and GT3a. The HCV near full-length amplification protocol was adapted to amplify both subtypes in the same reaction and the amplicon was submitted for next generation sequencing with the MiSeq 2 × 300 PE chemistry. Analysis of the aligned reads identified two populations, a GT1a and a GT3a population. The presence of two HCV viral populations is shown in a coverage plot stratified by genome position

Due to the transmission dynamics of HCV, multiple infections can occur concurrently producing a “co-infection” with two or more HCV genotypes present at a single time point. In the cohort used in this study the multiple infection rate has been previously reported to be as high as 25 % [17]. We therefore selected two known multiple infection samples, a GT1a/3a and a GT1a/2b sample (HITP300345 and HITP300315, respectively). We multiplexed the reverse primers at equimolar concentrations to test if both genotypes could be simultaneously amplified in the same nRT-PCR reaction. To confirm that this was successful the single amplicon was sequenced on the Illumina platform and the sequence data aligned against both the GT reference sequences (Fig. 4). Sequence reads were successfully mapped to both genotypes from the same sample. The data for subject HITP300345 is shown in Fig. 4, where 29.6 % and 70.4 % of sequence reads aligning to GT1a and 3a, respectively. Consensus sequences for both genotypes identified in each of the two subjects, HITP300345 and HITP300315 were generated and were clearly shown by phylogenetic analysis to belong to two separate genotypes (Fig. 1). The potential of this assay to be used to quantify mixed infection was not assessed. We believe that differences in genotype-specific PCR efficiency due to PCR primer bias and differences in secondary RNA structure would likely result in a semi-quantitative output. In order to quantify co-infections, a RT-PCR targeting a smaller region and with higher sensitivity would be more reliable [17]. The advantage of this assay is that it describes a pipeline for a cost efficient method to obtain full-genome sequences for phylogenetic and SNP analysis without the need to duplicate PCR assays for multiple genotypes in a single infection.

## Conclusions

In this study we have described a robust assay that can amplify the near full-length genome from all six major HCV GTs. The method can also be simply adapted to detect and sequence multiple infection. The ability to amplify the full genome in a single amplicon as opposed to multiple fragments reduces upstream cost and labour for multiple PCR reactions. Furthermore this method can be easily applied to sequence via multiple platforms, including Sanger sequencing, single genome amplification, short read deep sequencing such as the Illumina and Roche platforms, or the long read platforms, such as PacBio. As the sequence read technology continues to improve, the near-full length sequence data will improve analyses across an array of virological interests, including the ability to perform in-depth within-host evolutionary analysis and also to look at linkage between emerging antiviral resistant sites.

## Availability of supporting data

The GenBank accession numbers for the near-full length genome Illumina generated consensus nucleotide sequences of the samples in Fig. 1 are as follows: KU871276 to KU871311, KJ437295, KJ437300 and KJ437342.

## Additional file

Additional file 1: Figure S1. Successful amplification of near full-length HCV genomes from multiple genotypes. Amplicons (~9.2 kb) were run on an agarose gel (0.8 %) and visualized on a Gel Doc molecular imager (Bio-Rad); M represents the DNA marker HyperLadder 1 (Bioline). Lanes 1 to 8 represent GT1a amplicons. Lanes 9 to 11 represent GT1b amplicons. Lanes 12 to 16 represent GT3a amplicons. All amplicons were purified and successfully sequenced on the Illumina platform, including the faintly visible band in lane 7. (PDF 2448 kb)

## Abbreviations

DAA: direct acting antiviral
GT: genotype
HCV: hepatitis C virus
NGS: next generation sequencing
NS: non-structural
PacBio: Pacific Biosciences
PCR: polymerase chain reaction
RT: reverse transcription
SMRT: single molecule real time
