# Supplementary figures and images for: A method for near full-length amplification and sequencing for six hepatitis C virus genotypes
Source: BMC Genomics. 2016 Mar 17;17:247. doi: 10.1186/s12864-016-2575-8 (PMC4797172; doi:10.1186/s12864-016-2575-8)

(kbp) M 1 2 3 4 5 6 7 8 9 10 11 12 13 14 15 16 M

10 —

1 —

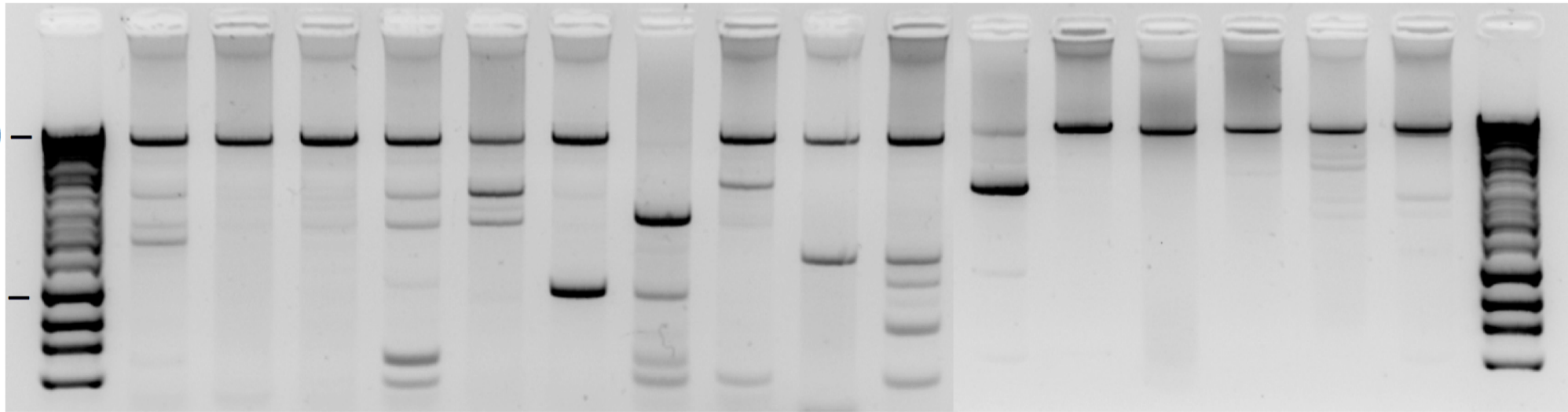

Supplement: Additional file 1: Figure S1. — Successful amplification of near full-length HCV genomes from multiple genotypes. Amplicons (~9.2 kb) were run on an agarose gel (0.8 %) and visualized on a Gel Doc molecular imager (Bio-Rad); M represents the DNA marker HyperLadder 1 (Bioline). Lanes 1 to 8 represent GT1a amplicons. Lanes 9 to 11 represent GT1b amplicons. Lanes 12 to 16 represent GT3a amplicons. All amplicons were purified and successfully sequenced on the Illumina platform, including the faintly visible band in lane 7. (PDF 2448 kb) [file 12864_2016_2575_MOESM1_ESM.pdf]
